# Phenotypic and genetic characterization of hypervirulent *Klebsiella pneumoniae* in patients with liver abscess and ventilator-associated pneumonia

**DOI:** 10.1186/s12866-023-03022-5

**Published:** 2023-11-13

**Authors:** Mingquan Guo, Bo Gao, Jun Su, Yigang Zeng, Zelin Cui, Haodong Liu, XiaoKui Guo, Yongzhang Zhu, Beiwen Wei, Yanan Zhao, Juanxiu Qin, Xiaoye Lu, Qingtian Li

**Affiliations:** 1 Department of Laboratory Medicine, Shanghai Public Health Clinical Center, Fudan University, Shanghai, China; 2 Shanghai Institute of Phage, Shanghai Public Health Clinical Center, Fudan University, Shanghai, China; 3 Department of Critical Care Medicine, Shanghai Pudong Hospital, Fudan University Pudong Medical Center, Shanghai, China; 4 Department of Laboratory Medicine, Shanghai General Hospital, Shanghai Jiao Tong University School of Medicine, Shanghai, China; 5 School of Global Health, Chinese Center for Tropical Diseases Research, Shanghai Jiao Tong University School of Medicine, Shanghai, China; 6 Department of Laboratory Medicine, College of Health Science and Technology, Shanghai Jiao Tong University School of Medicine, Shanghai, China; 7 Department of Laboratory Medicine, Renji Hospital, Shanghai Jiao Tong University School of Medicine, Shanghai, China; 8 Department of Emergency Medicine, Renji Hospital, Shanghai Jiao Tong University School of Medicine, Shanghai, China

**Keywords:** Hypervirulent *K. pneumoniae*, Ventilator-associated pneumonia, Pyogenic liver abscess, Biomarkers, Klebrate tool

## Abstract

Ventilator-associated pneumonia (VAP) and pyogenic liver abscess (PLA) due to *Klebsiella pneumoniae* infection can trigger life-threatening malignant consequences, however, there are few studies on the strain-associated clinical pathogenic mechanisms between VAP and PLA. A total of 266 patients consist of 129 VAP and 137 PLA were included for analysis in this study. We conducted a comprehensive survey for the two groups of *K. pneumoniae* isolates, including phenotypic experiments, clinical epidemiology, genomic analysis, and instrumental analysis, i.e., to obtain the genomic differential profile of *K*. *pneumoniae* strains responsible for two distinct infection outcomes. We found that PLA group had a propensity for specific underlying diseases, especially diabetes and cholelithiasis. The resistance level of VAP was significantly higher than that of PLA (78.57% vs. 36%, *P* < 0.001), while the virulence results were opposite. There were also some differences in key signaling pathways of biochemical processes between the two groups. The combination of *iucA*, *rmpA*, hypermucoviscous phenotype, and ST23 presented in *K. pneumoniae* infection is more important and highly prudent for timely treatment. The present study may contribute a benchmark for the *K. pneumoniae* clinical screening, epidemiological surveillance, and effective therapeutic strategies.

**Supplementary Information:**

The online version contains supplementary material available at 10.1186/s12866-023-03022-5.

## Background

*Klebsiella pneumoniae* is a common clinical pathogen that can cause community-acquired infections like urinary tract infections and pneumonia, but also invasive infections including bloodstream infections, pyogenic liver abscess (PLA), endophthalmitis, and meningitis [1, 2]. It can be divided into classic *K. pneumoniae* (cKp) and hypervirulent *K. pneumoniae* (hvKp) based on their pathogenic features [3]. As a new emerging scourge for public health, hvKp frequently manifests as acute onset, rapid progress, and systemic disseminated infections, and also occurs in immunocompetent and healthy individuals [4, 5]. In contrast to cKP, which commonly cause community-acquired infections, hvKp is associated with severe complications leading to high morbidity and mortality [6, 7]. It is generally accepted that hvKp is highly virulent rather than highly resistant, but in recent years, the detection rate of both highly pathogenic and multidrug-resistant hvKp is increasing, particularly carbapenem-resistant hvKp (CR-hvKp) [8–10]. Thus, it is critical to grasp their features in order to make an accurate clinical diagnosis and manage this alarming clinical condition effectively [3].

Similarly, ventilator-associated pneumonia (VAP) is associated with multi-drug resistance (MDR), high mortality, prolonging intensive care unit (ICU) stay and increasing healthcare costs. Gram-negative pathogen associated VAP accounted for the majority, with *K. pneumoniae* accounting for 11.9–37% [11, 12]. Severe pneumonia caused by hvKp infection is the most troublesome clinical problem, which often accompanied by co-infection or systemic disseminated infection, leading to severe disabling and fatal events [13, 14].

Many efforts have been devoted to unveiling the pathogenic properties of hvKp, multilocus sequence typing (MLST) and capsular antigen serotypes have also been used to identify hvKp generally [15, 16]. Some sequence types (such as ST23, ST65 and ST86) and capsular serotypes (such as K1 and K2) are closely associated with hvKp, especially ST23, ST86 are significantly associated with PLA, but these genotypes and serotypes are also present in VAP isolates [17, 18]. The hypervirulent phenotype of hvKp is thought to be attributable to the carriage of a set of virulence factors like capsular polysaccharide (CPS) regulator (*rmpA1/2*) genes, some key virulence determinants on a plasmid (such as *peg-344*, *iroBCDN*, *iucABCD*, etc.), aerobactin and several siderophore gene clusters, but none of these is specific to hvKp [2, 19]. So far, some virulence factors like capsular polysaccharides, lipopolysaccharides, and adhesins were known to be closely related to the virulence of hvKp [3, 20], however, the biomarker specified by a combination of virulence factors that determines the pathogenicity of hvKp is still unclear [21].

Pathogenic mechanisms of hvKp infection are multifactorial, with type 2 diabetes (T2D), dietary habits, and gut microbiota composition being key host factors [22]. Still, routine in vitro tests such as string test, MLST, virulence factor screening, neutrophil phagocytosis/killing assay, and even *Galleria Mellonella* infection model has not been validated enough to provide a reliable reference for clinical prediction of bacterial pathogenicity [23–25]. Thus, a better understanding of the underlying mechanism of hvKp infection is important for promoting recovery and reducing the occurrence of complications.

In this study, we systematically performed a preliminary investigation of the genetic features for differentiation of hvKp infection between VAP and PLA from Shanghai Pudong Hospital, Fudan University Pudong Medical Center. This is a rare study of a comprehensive comparative analysis of *K. pneumoniae* isolates from VAP and PLA cases, including bronchoalveolar lavage (BAL), blood, sputum, puncture and drainage samples. Therefore, this may provide reasonable and scientific prospective evidence on the discrimination of genotypic and phenotypic biomarkers for these two malignant infections.

## Results

### Demographics, disease course, and outcome of patients involved in this study

**Fig. 1:**
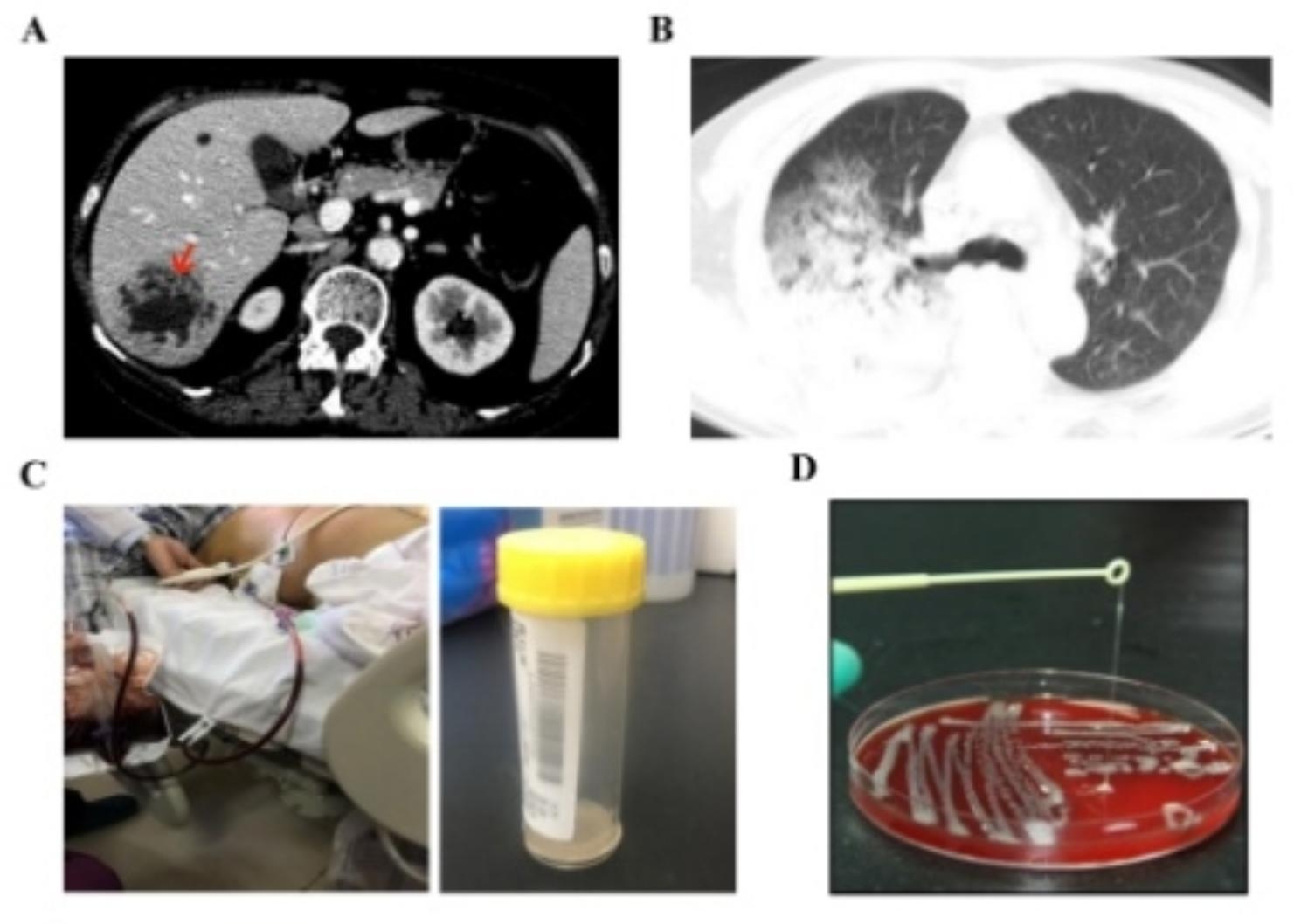
Schematic presentation of *K. pneumoniae* investigated in VAP and PLA patients. X-ray computed tomography (CT) was employed to obtain the radiography pictures. (**A**) Representative radiography pictures of invasive liver abscess (red arrow) form a PLA patient. (**B**) Lung imaging pictures of patients with ventilator-associated severe pneumonia. (**C**) Clinical samples including puncture fluid. (**D**) Clinical microbiology laboratory test

Among the 266 patients, 129 VAP patients and 137 PLA patients were included in this study. According to our analysis, the proportion of males in both groups was slightly higher than that of females. The average age in the two groups showed significant differences, patients with VAP were 59.52 ± 14.36 years, 65.47 ± 13.45 in PLA. Culture positive *K. pneumoniae* was found in VAP group (26/126, 20.16%) and PLA group (34/137, 24.82%). And the majority of patients with VAP are respiratory department (12/26, 46.15%) and ICU (11/26, 42.30%), and most of the clinical isolates obtained late in the development of infection due to the state of ventilator use. In all, fourteen patients with VAP and twenty-five patients with PLA were included for analysis. The hospital stay time of VAP group was 46.32 ± 24.25 days, and that of PLA group was 17.21 ± 8.42 days (*P* < 0.001). There was a significant difference in mortality between two groups, with four deaths in the VAP group and one death in the PLA group (Table 1). There was significant difference between two groups in terms of underlying disease, especially for diabetes and cholelithiasis (*P* < 0.001). As shown in Fig. 1A, medical imaging analysis of liver abscess caused by hvKp infection revealed liver cavities and massive pleural effusion infiltration, i.e., lungs of patients with severe pneumonia showed multiple patchy consolidations and delayed opacity, and partial air space infiltration (Fig. 1B). The patient was aspirated by puncture drainage of pus to separate the sample (Fig. 1C). By performing a string test on these isolates (Fig. 1D), we determined that there were 22 (of 25, 88%) positive isolates in the PLA group and 5 (of 14, 35.71%) in the VAP (*P* < 0.001).

**Table 1:** The demographic and clinical characteristics between patients with the severe pneumonia and pyogenic liver abscess

Demographics	Strains cohorts		*P* Value
	PLA (n = 25)	VAP (n = 14)	
Age (y, mean ± SD)	53.31 ± 17.54	67.86 ± 23.13	< 0.001
Genders (male)	16 (64%)	8 (57.14%)	
Genders (Female)	9 (36%)	6 (42.86%)	
Comorbidity			
Renal insufficiency	4 (16%)	5 (35.71%)	
Diabetes mellitus	9 (36%)	3 (22.22%)	< 0.001
Biliary tract disease	7 (28%)	0	< 0.001
Cholelithiasis	5 (20%)	1 (7.14%)	
Smoking history	5 (20%)	4 (28.57%)	
Hypertension	2 (8%)	5 (35.71%)	
Respiratory insufficiency	1 (4%)	11 (78.57%)	
Septic pulmonary embolism	2 (8%)	5 (35.71%)	
Co-infection	0	6 (42.85%)	< 0.001
Hospital stays (day)	17.21 ± 8.42	46.32 ± 24.25	< 0.001
Deceased	1 (4.34%)	4 (23.53%)	< 0.001

### Antimicrobial susceptibility of isolates

In Table 2, we found that the antibiotic resistance rate of *K. pneumoniae* in the VAP group was significantly higher than that in the PLA group (*P* < 0.001). MDR isolates in VAP were 11 (of 14, 78.57%), while in PLA were 12 (of 25, 48%). The resistance rate in two groups, the highest resistance rate was for ampicillin (100%). In PLA group, only ampicillin, ciprofloxacin, and cefepime had resistance rate of more than 50%, those with resistance rate less than 30% were for amikacin (28%), piperacillin-tazobactam (24%), imipenem (0%) and meropenem (4%), respectively. In the VAP group, only four antibiotics had resistance rate below 50%, including tigecycline (0%), amikacin (42.85%), and imipenem (21.24%) and meropenem (28.57%). There was one polymyxin B-resistant strain in the VAP group, while fully sensitive in the PLA group.

**Table 2:** Comparison of drug resistance patterns between two groups of *K. pneumoniae*

Antimicrobial agents	Strains cohorts		*P* Value
	PLA	VAP	
Hypermucoviscosity	22 (88%)	6 (35.71%)	< 0.001
MDR	12 (48%)	11 (78.57%)	< 0.001
Ampicillin	25 (100%)	14 (100%)	
Ciprofloxacin	17 (68%)	10 (71.29%)	
Amikacin	7 (28%)	6 (42.85%)	< 0.001
Cefoperazone/sulbactam	11 (44%)	10 (71.43%)	< 0.001
Piperacillin/tazobactam	6 (24%)	9 (57.14%%)	< 0.001
Cefazolin	15 (60%)	5 (35.71%)	
Ceftazidime	14 (48%)	11 (78.57%)	
Cefepime	15 (60%)	10 (71.43%)	
Cotrimoxazole	9 (36%)	11 (78.57%)	< 0.001
Imipenem	0	3 (21.24%)	< 0.001
Meropenem	1 (4%)	4 (28.57%)	< 0.001
Polymyxin B	0	1 (7.14%)	
Tigecycline	0	0	

### Comparison of virulence and drug resistance scores between the VAP and PLA isolates

**Fig. 2:**
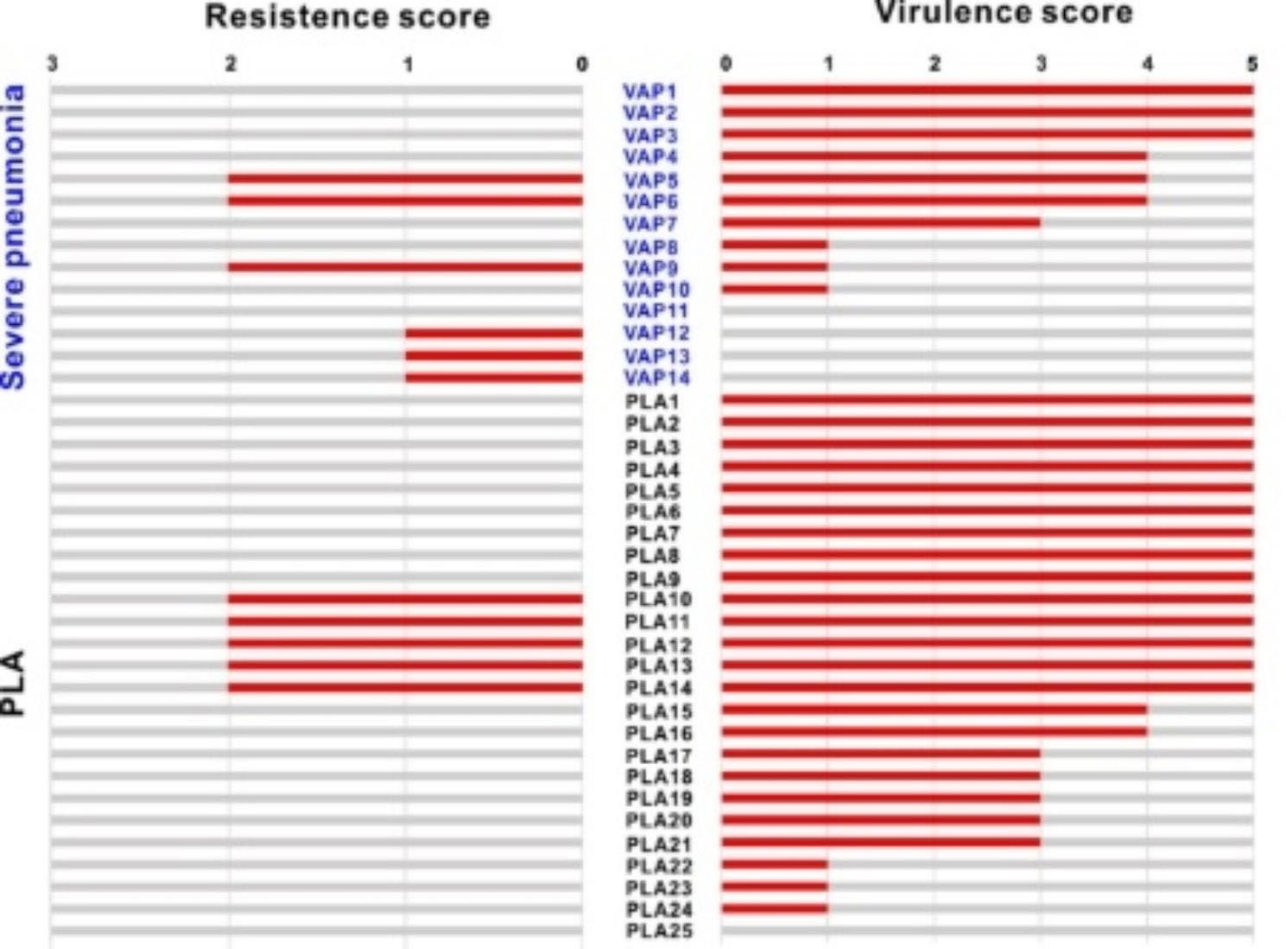
Distribution of resistance and virulence scores of genomes between PLA and VAP *K. pneumoniae* isolates. Resistance and virulence scores of genomes were demonstrated by Kleborate tool. For resistance score, from 0 to 3, 0: ESBL^−^, Carb^−^, 1: ESBL^+^, Carb^−^, 2: Carb^+^, 3: Carb^+^, Col^+^. For virulence score, 0: None, 1: *ybt*, 2: *ybt* + *clb*, 3: *iuc* (VP), 4: *ybt* + *iuc* (VP), 5: *ybt* + *clb* + *iuc* (VP). The blue fonts for VAP strains

Histograms showed the virulence and resistance scores of *K. pneumonia*e isolates (Fig. 2). Compared with these isolates in the VAP group, the PLA isolates exhibited higher antibiotic sensitivity. Only 20% (5/25) of the isolates in the PLA group had resistance scores more than 1, while 42.85% (6/14) in the VAP group (*P* < 0.001). In particular, *carb*^+^ gene distribution was significantly more in the VAP group than the PLA group (*P* < 0.001). The virulence scores showed that the PLA group had a much greater distribution of genomic virulence determinants than that in VAP. With the virulence score of more than 3 in the PLA group accounted for 21 (of 25, 84%), compared with 7 (of 14, 50%) in VAP group. The PLA group with virulence score of 0 was only 1 (of 25, 4%) isolate, while 4 (of 14, 28.57%) isolates in VAP group. Also, it is interesting to note that additional extended-spectrum β-lactamase (ESBL)-positive isolates showed susceptibility to cefepime in our result, suggesting that other potential resistance mechanisms should be considered while interpreting susceptibility.

### ST, Resistance and virulence profiles of *K. pneumoniae* isolates

**Fig. 3:**
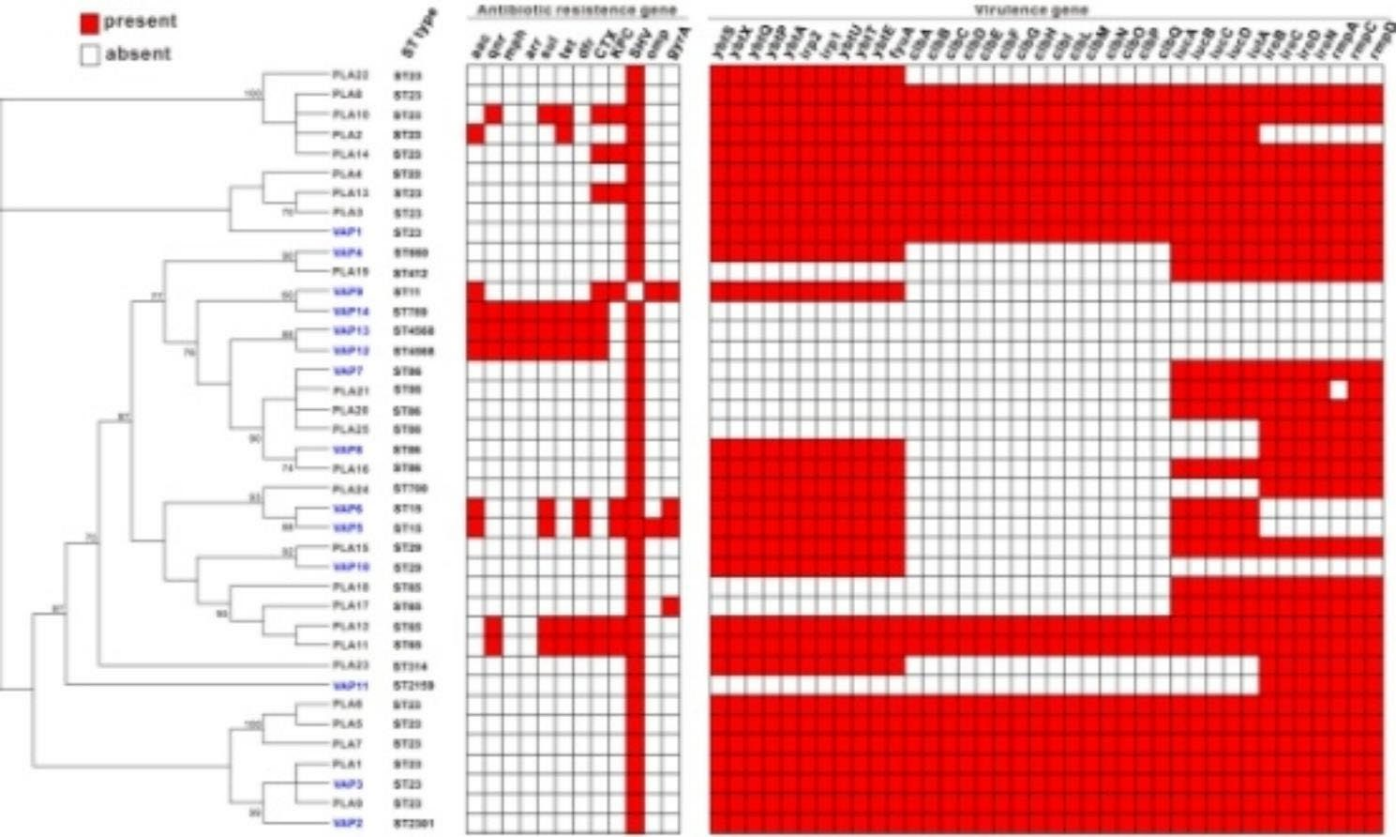
Phylogenetic characteristics, resistance/virulence gene patterns of *K. pneumoniae* isolates between PLA and VAP. MLST typing, resistance virulence gene screening of isolates were analyzed by Kleborate tool. Squares shaded with red background means the presence of genes, and blue fonts for VAP strains

PLA isolates comprised seven ST types (Table S1-2), with the majority belonging to ST23 (13/25, 52%), ST65 (4/25, 16%), and ST86 (4/25, 16%). The isolates of VAP patients consisted of nine STs, and the predominant subtype of which was also ST23 (3/14, 21.43%), followed by ST15, ST29, and ST86 (2/14, 14.29%). Comparative genomic analysis of the isolates showed that the two groups of strains could be divided into five different evolutionary clades, and no substantial difference was found between the two groups (Fig. 3).

The Kleborate analysis found that β-lactamase SHV-type resistance genes were present in almost all of the strains. And *aac*, *mph*, and *arr* genes were found to be considerably more abundant in VAP strains than in PLA strains. No resistance gene was discovered to be considerably more prevalent in the PLA group than in VAP. Combined analysis of virulence genes showed that the virulence gene pattern of isolates in the PLA group was higher than the VAP group. Virulence factors *iuc* (aerobactin), *iro* (salmochelin), and *rmpA* were more widely distributed in the PLA strains, although *ybt* (yersiniabactin), *fyu* and *clb* (cyclopropane hydrolase) genes were not substantially different between the two groups. Meanwhile, we found that the virulence genes of ST23 and ST65 strains were more widely distributed compared to other ST subtype, and these two types were the dominant type in the PLA group. Similarly, we found significant differences in serological analysis between the two groups, serotype K1 predominated in both groups, PLA (14/25, 56%) vs. VAP (4/14, 29%), followed by serotype K2, PLA (8/25, 32%) vs. VAP (4/14, 14%). K23, K54, and K57 were also present in 4% of the PLA group. K16 (7%), K18 (7%), K19 (14%), K54 (14%), and K127 (14%) were present in the VAP group.

### Functional genome analysis of the VAP and PLA *K. pneumoniae* isolates

**Fig. 4:**
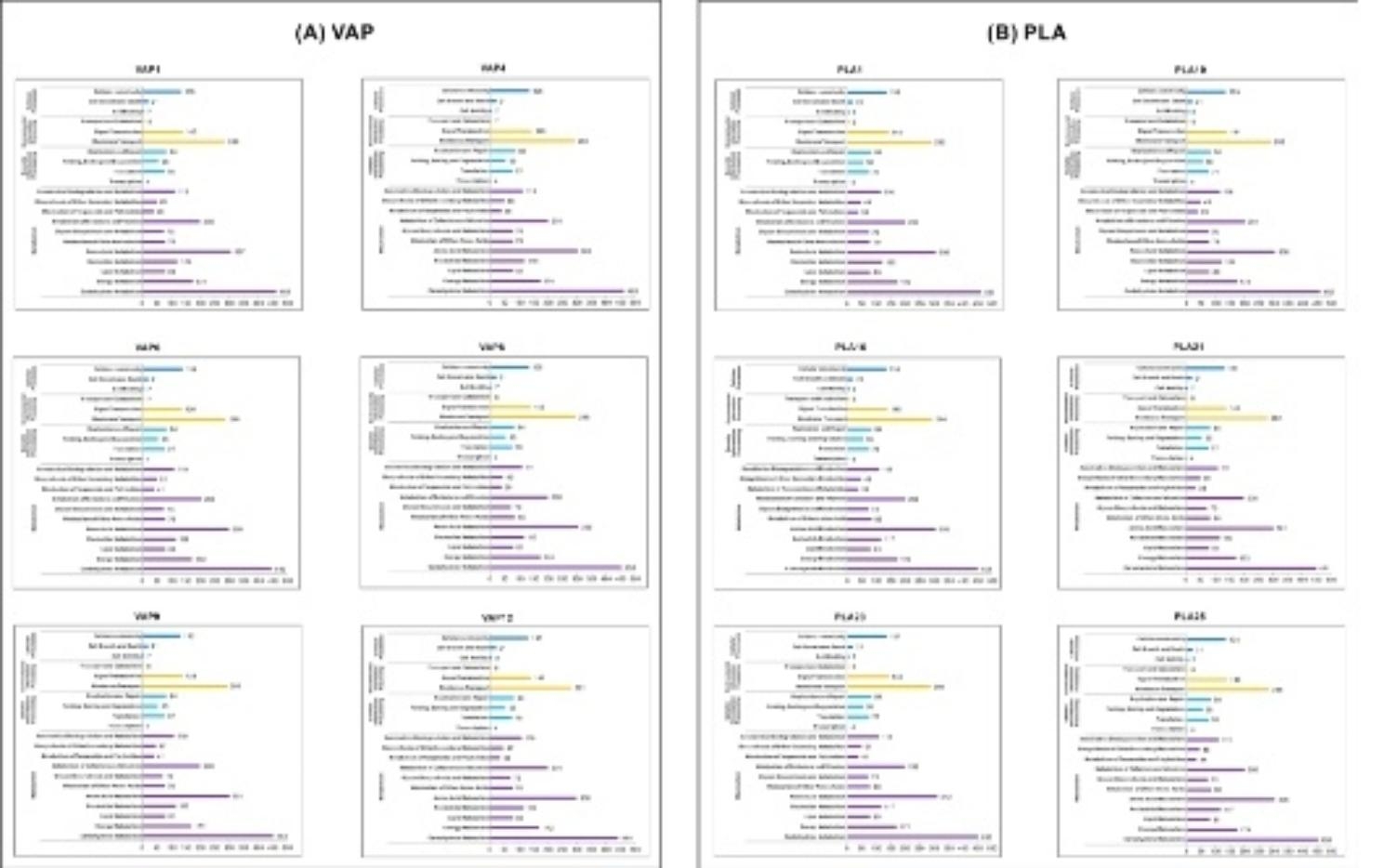
Comparative analysis of key signaling pathways in biochemical processes of isolates. Representative isolates from the PLA and VAP groups were selected to map key signaling pathways of biochemical processes of *K. pneumoniae*. CorelDRAW X8 was used to perform combinatorial mapping to present the functional gene distribution of key signaling pathways in different isolates genome. (*A*) VAP isolates. (*B*) PLA isolates

Six PLA and VAP representative isolates were selected to map the key signaling pathways of biochemical processes in key signaling pathways between different strains (Fig. 4). Generally, the key metabolic pathway files differed slightly between PLA and VAP groups. PLA strains revealed more active amino acid metabolism, while within-group comparison showed a more concentrated distribution of amino acid metabolism genes in the VAP group. Metabolic pathways of lipids and vitamins were found to be not significantly different between VAP and PLA groups. However, it can be noted that the membrane transport system was more abundant in the VAP group strains than in PLA. Through the deep mining of metabolic function genes, in a comparison of genomic cellular transport and catabolism signaling pathways between strains of different virulence scores, such as VAP1, PLA1, and PLA10 (virulence score of 5) strains, and VAP8, VAP9, PLA21, PLA23, and PLA25 (virulence score of less than 3), it was found that hypothetical protein MS64 and beta-mannosidase may be positively correlated with strain virulence.

## Discussion

*K. pneumoniae* is a serious threat to public health. The genomic properties of *K. pneumoniae* make it a priority for the World Health Organization (WHO), with the consequences of bacterial resistance leading to poorer patient outcomes and higher risk of treatment costs and death [26, 27]. As reported in previous epidemiological studies of hvKp, we found males with biliary disease and diabetes were at increased risk of hvKp infection [28, 29]. The plausible explanation for *K. pneumoniae* PLA of diabetic patients is that the high glucose environment leads to impaired host immunity while enhancing the ability of hvKp to synthesize capsular polysaccharides [24]. Patients with biliary tract disease as the underlying disease may be associated with the dissemination of hvKp bacteria in the patient’s gut [30]. In contrast, the infection propensity of *K. pneumoniae* in VAP differed from that in PLA in previous studies [31]. We found that the VAP group had significantly longer hospital stays and higher mortality compared to the PLA group, which may be mainly due to the limitations of the choice of antibiotics available for patients.

Combined with the results of previous epidemiological surveys of PLA patient cohort in China, the results of genotype analysis showed that ST23 and ST86 still were the dominant strains in the invasive liver abscess [29, 32]. And then, ST11, ST258 strains were closely related to drug resistance and often appear in pneumonia [33], ST23, ST15, and ST29 were the dominant isolates in VAP group in this study. In addition, capsular serotypes such as K1 and K2 are strongly associated with high virulence, as we found in our study, they were dominant serotypes for PLA strains. There is increasing evidence from animal experiments to support that the hypermucoviscosity phenotype can somehow describe the hypervirulent phenotype of *K. pneumoniae* [34], we also found that the hypermucoviscosity phenotype of *K. pneumoniae* in PLA is significantly higher than that of VAP.

The detection rate of MDR *K. pneumoniae* has recently aroused increasing attention, especially ESBL-producing and carbapenem-resistant isolates [35]. In the present research, ESBL-resistant and carbapenem-resistant isolates in VAP were higher than PLA group (~ 30% vs. ~ 4%), which revealed that antimicrobial resistance is on the rise in both cKP and hvKp strains [36]. Hypothetical mechanisms include that VAP isolates colonize the medical device and environment continuously and gradually mutate into MDR strains under antibiotic pressure, while the majority of PLA strains can be effectively killed by antibiotic treatment at the initial stage of infection [37, 38]. Based on the difference in the proportion of hvKp strains between the two groups, it has been speculated that the rapid acquisition of the determinants of antibiotic resistance, plasmid integration, the physical barrier of polysaccharide capsules, spaced short palindromic repeats (CRISPR) systems may be responsible [33].

It is also important to note that until now, given the complexity of phenotypic predictions including string test experiments and in vivo animal tests were unable to accurately reveal the underlying genetic mechanisms of hvKp strains [23, 39]. And then, there has been even less research aimed at distinguishing KP-VAP from KP-PLA. Previous studies have reported that multiple biomarkers such as *peg-344*, *rmpA*, *iucA* and *iroB* could be used as accurate genetic markers to distinguish this pathotype of hvKp from cKp to some extent [21]. In this study, we found that *iucA*, one of the genes in the *iuc* operon encoding aerobactin, a critical mediator of virulence, and it is significant differences in genome distribution between the VAP and PLA strains, although the strains studied were somewhat different in cKp and hvKp [40, 41]. Secondly, *rmpA*, a key virulence factor of regulators of the mucoid capsule synthesis, played a key role in distinguishing VAP from PLA group isolates, which has the effects of anti-neutrophil and macrophage phagocytosis and inhibiting dendritic cell maturation. Indeed, previous studies in mouse infection experiments have shown that, *rmpA* acts as a key virulence factor, and overexpression of RmpA in cKP isolates enhanced the bacterial virulence, presenting a hypervirulent phenotype [42]. As we analyzed in this study, serotype K1 accounted for a much higher proportion of strains in the PLA group than in VAP, and virulence determinants showed a similar distribution.

Invasion and toxins constitute bacterial virulence, and their material basis is the bacterial structure and metabolites of bacteria [7]. We compared the key signaling pathways of *K. pneumoniae* isolates between PLA and VAP for the first time, and we found that membrane transport system signals were more abundant in VAP strains than PLA. We hypothesized that perhaps this phenomenon explains, to some extent, the complexity of VAP strains in the resistance mechanism. Based on the in-depth mining of functional genes in key metabolic pathways, we speculated that MS64 and β-mannosidase, key factors related to phagocytosis and lysosomes on the hvKp genome, play a key role in determining bacterial virulence. Taken together, there are still some shortcomings in this study, that is, the relevant study data have limited isolates, and further studies should be performed to verify the range of isolates with different clinical outcomes.

## Conclusions

We systematically performed a preliminary assessment of the key genetic features for differentiating of hvKp infection between VAP and PLA. The combination of *iucA*, *rmpA*, hypermucoviscous phenotype, and ST23 presented in *K. pneumoniae* infection is of more concern. In an era of increasing drug resistance and relatively stagnant antimicrobial drug development, these findings can facilitate a rapid and effective clinical treatment response to avoid the deadly nosocomial outbreaks for this superbug.

## Methods

### Subjects and data collection

This study was conducted retrospectively in Pudong Hospital from January to December 2020. A total of 266 patients from ICU, emergency department and respiratory department were included in this study. Inclusion criteria, two cohorts of patients infected with *K. pneumoniae* isolates including VAP patients with severe pneumonia and PLA were randomly selected for analysis according to different admission time and wards, and the clinical symptoms and groups were strictly identified by senior experts from the department of infectious diseases. Exclusion Criteria: patients with non-*K. pneumoniae* pathogens. All clinical data information including clinical manifestations, laboratory examination, ultrasound, magnetic resonance imaging (MRI) data, treatment plan, and outcome during hospitalization were obtained from the computerized database in the hospital.

## Laboratory clinical microbiological examination

Colombia Blood Plates (Bobio Biotechnology Co., LTD., China) were used to isolate bacteria from sputum, blood, BAL and drainage fluid. Culture method was conducted at 35 ℃, 5% CO_2_ incubator for 16 to 18 h, and with smears and Gram staining concurrently according to the standard operating procedures of the clinical microbiology laboratory. The Microflex matrix-assisted laser desorption ionization time-of-flight (MALDI-TOF) mass spectrometry (Bruker Daltonics Inc., Germany) and VITEK® 2 GN Compact system (BioMérieux, Lyon, France) were used for the authentication for the culture-positive isolates. All the strains were stored and retrieved at -80 ℃ strictly following the Regulations on the Biosafety Management of Pathogenic Microbiology Laboratories issued by the State Council of the People’s Republic of China to ensure effective and safe use.

### Hypermucoviscosity phenotype and antimicrobial susceptibility

String test was used to detect mucoid phenotype of isolates following the routine method [43]. Kirby-Bauer disc (KB) diffusion (Thermo Fisher Scientific, Waltham, MA, USA) and VITEK® 2 AST-GN13 (BioMérieux, Hazelwood, France) were used for the antimicrobial susceptibility detailed by the Clinical and Laboratory Standards Institute Guidelines (CLSI-2019). The results were interpreted according to CLSI guidelines, AST-GN13 cards including Amp, ampicillin, Sam, ampicillin/sulbactam; Amk, amikacin; Tms, trimethoprim–sulfamethoxazole; Azm, aztreonam; Ctn, cefotetan; Cax, ceftriaxone; Cfz, cefazolin; Cpe, cefepime; Cp, ciprofloxacin; Caz, ceftazidime; Gm, gentamicin; To, tobramycin; Lvx, levofloxacin; Imp, imipenem; Etp, ertapenem; Ptz, piperacillin/tazobactam. *Escherichia coli* ATCC25922 was used as reference strain. And the interpretive criteria of KB test for tigecycline (TGC), Imipenem (IMP) and meropenem (MEM) was categorized based on the European Committee on Antimicrobial Susceptibility Testing (EUCAST) and U.S. Food and Drug Administration (FDA) susceptibility breakpoints for Enterobacteriaceae [44]. Polymyxin B drug sensitivity kit (Bio-KONT® Biotechnology Co., LTD., China) was performed by Microbroth dilution method, and *E. coli* ATCC 25,922 and *Pseudomonas aeruginosa* ATCC 27,853 were used as controls.

### Genome sequencing, assembly, and functional annotation

Genomic DNA from these isolates was routinely extracted using the QIAamp DNA Mini Kit (Qiagen® 51,304, Germany), Quality evaluation and DNA quantification were carried out with the Ultrafine UV spectrophotometer NanoDrop™ One/OneC (Thermo Fisher Scientific Inc., USA), DNA concentration > 100 ng µL^− 1^. The TruSeq™ DNA Sample Prep Kit (Illumina) was used to construct a 450-bp sequencing library according to the default instructions. Draft genome sequencing was conducted by the Illumina NovoSeq platform in Shanghai Personal Company. The sequencing adaptors and low-quality reads were trimmed and filtered using Fastp (https://github.com/OpenGene/fastp). Subsequently, *De novo* genome assembly was carried out using Shovill v1.1.0 with default options. The assembly statistics and average nucleotide identity of different assemblies were evaluated using Quast v5.0.2 (https://github.com/ablab/quast). By a combination of publicly available complete *K. pneumoniae* genome as a local database, these newly sequenced genomes were annotated using the pipeline Prokka v1.4.5.

### Bioinformatic analysis

Online taxonomy identification was performed using PathogenFinder 1.1 (https://cge.cbs.dtu.dk/services/PathogenFinder/). Virulence factors and antimicrobial resistance (AMR) genes were identified by the VFDB database (http://www.mgc.ac.cn/cgi-bin/VFs/v5/main.cgi) and ResFinder 4.0 (https://cge.cbs.dtu.dk/services/ResFinder/), respectively. Pan-genome analysis was carried out using the Roary pipeline with the parameters being -e, -n and a minimum percentage identity of 90%. The binary matrix with the presence and absence of each gene across all strains was used to estimate the sizes of the pan-genome and core-genome by the program PanGP v1.0.1. The protein sequences were functionally annotated and classified by multiple databases including KEGG, COG, and SwissProt. The key signaling pathways of *K. pneumoniae* biochemical processes was analyzed, such as metabolism, membrane transport, signal transmission of genetic and environmental information processing, and cell cycle, respectively. CorelDRAW Graphics Suite (Version X8) was used to perform combinatorial mapping to present the functional gene distribution of key signaling pathways in different genomes.

To better quantify the distribution of determinants of drug resistance and virulence on the genome of isolates, Kleborate tool (v.2.0.0) were used to determine MLST, antigen prediction, virulence factors (*rmpA*/*rmpA2*, *iroBCDN*, *iucABCD*, *iuc*, *iutA*, *peg-344*, *ybt*, etc.) and resistance genes (*aadA2*, *dfrA12*, *CTX-M*, *mphA*, *ermB*, *sul1*, *rmtF*, *qnrS1*, *dfrA12*, *strA*, etc.) (http://github.com/katholt/Kleborate). Virulence and AMR scores were analyzed by Kleborate summary calculation of locus accumulation for resistance and virulence [45]. Resistance scores range from 0 to 3, based on the presence of extended-spectrum β-lactamase (ESBL), carbapenemase (Carb), colistin resistance genes (Col). For resistance score, from 0 to 3, 0: ESBL^−^, Carb^−^, 1: ESBL^+^, Carb^−^, 2: Carb^+^, 3: Carb^+^, Col^+^. Virulence scores range from 0 to 5, depending on the presence of key loci such as *ybt*, *clb* (colibactin), *iuc*, etc. [45]. For virulence score, 0: None, 1: *ybt*, 2: *ybt* + *clb*, 3: *iuc* (VP), 4: *ybt* + *iuc* (VP), 5: *ybt* + *clb* + *iuc* (VP).

### Statistical analysis

Statistical analyses in this study were expressed as Mean ± SD as indicated. F test was used to determine the equality of variances between VAP and PLA groups for parametric analysis, Student’s *t*-test was analyzed to continuous variables across the two groups, one-way analysis of variance (ANOVA) were performed to determine significant differences, *P* value *<* 0.05 was considered statistically significant.

### Electronic supplementary material

Below is the link to the electronic supplementary material.

Supplementary Material 1

Supplementary Material 2

Supplementary Material 3

## Data Availability

The nucleotide sequences of each strain genome. during the current study are available in the Chinese CNCB database repository (https://ngdc.cncb.ac.cn/gwh/), under accession number: PRJCA009406.

## Abbreviations

AMR: antimicrobial resistance
BAL: bronchoalveolar lavage
cgMLST: core genome multilocus sequence typing
cKp: classic *K. pneumoniae*
CLSI: Clinical and Laboratory Standards Institute
CPS: capsular polysaccharide
CR-hvKp: carbapenem-resistant hypervirulent *K. pneumoniae*
CT: computed tomography
ESBL: extended-spectrum β-lactamase
hvKp: hypervirulent *K. pneumoniae*
ICU: intensive care unit
MALDI-TOF: matrix-assisted laser desorption ionization time-of-flight
MLST: multilocus sequence typing
MRI: magnetic resonance imaging
PLA: pyogenic liver abscess
T2D: type 2 diabetes
VAP: ventilator-associated pneumonia
WHO: World Health Organization
