# Supplementary material for: Phenotypic and genetic characterization of hypervirulent Klebsiella pneumoniae in patients with liver abscess and ventilator-associated pneumonia
Source: BMC Microbiol. 2023 Nov 13;23:338. doi: 10.1186/s12866-023-03022-5 (PMC10644596; doi:10.1186/s12866-023-03022-5)
Supplement: Supplementary file 1 — Supplementary Material 1 [file 12866_2023_3022_MOESM1_ESM.pdf]

Supplementary Materials:

Table S1: Antimicrobial resistance (AMR) and virulence profile from Kleborate tool analysis for VAP strains.

Table S2: Antimicrobial resistance (AMR) and virulence profile from Kleborate tool analysis for PLA strains.
